# Investigation of astrovirus and bornavirus in the cerebrospinal fluid of dogs clinically diagnosed with meningoencephalitis of unknown etiology

**DOI:** 10.1111/jvim.15677

**Published:** 2019-11-30

**Authors:** Audrey Collinet, Gabriel Garcia, Jim Wellehan, April Childress, Sheila Carrera‐Justiz

**Affiliations:** ^1^ Department of Small Animal Clinical Sciences, College of Veterinary Medicine University of Florida Gainesville Florida; ^2^ Department of Comparative Diagnostic and Population Medicine, College of Veterinary Medicine University of Florida Gainesville Florida

**Keywords:** canine, inflammation, meningoencephalitis of unknown origin, meningoencephalomyelitis, virus

## Abstract

**Background:**

Non‐suppurative encephalitides in a variety of species, including humans and dogs, have been linked to infection by astroviruses and bornaviruses.

**Hypothesis/Objectives:**

To determine whether or not ribonucleic acid of astroviruses or bornaviruses was present in the cerebrospinal fluid (CSF) of dogs with clinically diagnosed meningoencephalomyelitis of unknown etiology (MUE).

**Animals:**

Twenty‐five client‐owned dogs evaluated by CSF analysis at a single university referral hospital.

**Methods:**

Prospective case‐control study. Cerebrospinal fluid was collected from clinically diagnosed MUE and control cases and evaluated by reverse‐transcriptase polymerase chain reaction for the presence of astrovirus and bornavirus.

**Results:**

Neither astrovirus nor bornavirus nucleic acids were identified in CSF collected from 20 clinically diagnosed MUE and 5 control cases.

**Conclusions and Clinical Importance:**

The negative results of this investigation suggest that astrovirus and bornavirus are not commonly detectable in CSF of dogs with MUE.

**Abbreviations**
CNS: central nervous system
CSF: cerebrospinal fluid
ESBE: European sporadic bovine encephalitis
FFPE: formalin‐fixed paraffin‐embedded
GME: granulomatous meningoencephalitis
MUE: meningoencephalomyelitis of unknown etiology
NLE: necrotizing leukoencephalitis
NME: necrotizing meningoencephalitis
RT‐PCR: reverse‐transcriptase polymerase chain reaction
TNCC: total nucleated cell count

## 1 INTRODUCTION

Meningoencephalomyelitis of unknown etiology (MUE) is the umbrella term applied to a common clinical diagnosis of noninfectious inflammatory central nervous system (CNS) disease. It most frequently encompasses granulomatous meningoencephalitis (GME), necrotizing meningoencephalitis (NME), and necrotizing leukoencephalitis (NLE), which are distinguished based on histopathologic characteristics. Efforts at identifying common etiologies for MUE have been unsuccessful. Etiologies that have been suggested include genetic, neoplastic, infectious, and autoimmune.^1^, ^2^, ^3^, ^4^, ^5^, ^6^, ^7^, ^8^

Immunohistochemical studies and response by MUE dogs to immunosuppression support an immune‐mediated pathogenesis.^2^ Viruses are of particular interest as factors priming the development of autoimmunity.^2^, ^9^ Previous veterinary studies evaluated for the presence of several neurotropic DNA viruses, RNA viruses, and atypical bacterial pathogens associated with encephalitis in humans by polymerase chain reaction (PCR), serology, culture, immunohistochemistry, and metagenomics sequencing.^1^, ^6^, ^7^, ^8^, ^10^ Two studies did not detect viruses in either fresh frozen or formalin‐fixed paraffin‐embedded brain tissue of dogs with MUE, although neither astroviruses nor bornaviruses were screened for in either study.^1^, ^6^

Non‐suppurative encephalitides have been linked to RNA viruses in the families Astroviridae and Bornaviridae, in a number of other species. Recently, encephalitis in cattle, mink, swine, sheep, and immunocompromised humans has been associated with astroviruses.^6^, ^11^, ^12^, ^13^, ^14^, ^15^, ^16^, ^17^, ^18^ Retrospective evaluation of brain samples from cattle with European sporadic bovine encephalitis (ESBE), a non‐suppurative polioencephalitis of previously undetermined etiology similar to MUE, confirmed the presence of astrovirus in 85% of samples.^18^ Bornavirus encephalitis was originally recognized in horses and sheep but has since been diagnosed in a diverse host range including birds and other reptiles, rabbits, cattle, cats, squirrels, humans, and 2 dogs.^19^, ^20^ It is unknown if bornavirus encephalitis is more widespread in dogs.

Immunosuppression of dogs with MUE is the current standard of care. Despite aggressive treatment, the course of disease is usually fatal. Identification of the underlying etiology of this disease complex is crucial to improve treatment plans and prognosis. The objective of this investigation was to evaluate using PCR‐based methods for the presence of astroviruses and bornaviruses in the CSF of dogs clinically diagnosed with MUE.

## 2 MATERIALS AND METHODS

### 2.1 Study population and sample collection

This study was approved by the University of Florida's Institutional Animal Care and Use Committee Study #201609667. As part of this prospective case‐control study, CSF was collected from client‐owned dogs evaluated at the University of Florida Small Animal Hospital Neurology Service for CNS disease from March 2016 through October 2018. All dogs underwent a complete physical and neurological examination by a neurology resident or board‐certified neurologist. Only dog breeds previously documented with GME, NME, or NLE were considered for inclusion. These included Terrier‐breed, Chihuahua, Brussels Griffon, Pug, French Bulldog, Papillon, Shih Tzu, Pekingese, Boston Terrier, Yorkshire Terrier, Cotton de Tulear, West Highland White Terrier, Poodle, Pomeranian, Dachshund, and Maltese or a cross of any of these breeds.^1^, ^2^, ^3^, ^5^, ^6^, ^21^, ^22^, ^23^, ^24^, ^25^ For inclusion, samples had to be collected from dogs diagnosed with MUE based on previously described guidelines.^24^ Specifically, a clinical diagnosis of MUE was considered in dogs older than 6 months of age with evidence of single, multiple, or diffuse intracranial or spinal lesions on MRI; a CSF pleocytosis (total nucleated cell count [TNCC] >5 cells/μL) with >50% mononuclear cells; and an absence of identifiable geographical infectious diseases. Regionally prevalent infectious diseases were excluded based on negative serum antibody titers to *Toxoplasma gondii*, *Neospora caninum*, and *Cryptococcus neoformans*. If necropsy results became available, these were reviewed for confirmation of MUE diagnosis.

Dogs were excluded from the study if they were identified as a breed other than those listed in the inclusion criteria or had positive serum titers for *Toxoplasma gondii*, *Neospora caninum*, or *Cryptococcus neoformans*. Samples were also excluded if the dog was diagnosed with an alternate CNS disease associated with a pleocytosis. Such diseases included CNS neoplasia, fibrocartilaginous embolism, and compressive myelopathy associated with intervertebral disc disease.^26^, ^27^

Dogs were considered as negative controls if they met the inclusion criteria, with the exception of the cerebrospinal fluid (CSF) analysis. Negative control samples were included if the TNCC was less than 5 cells/μL on CSF analysis.

A power analysis was done to determine sample size to detect the presence or absence of astroviruses or bornaviruses in dogs with MUE.^28^ An average of 33 cases are diagnosed with MUE by the University of Florida's Neurology Department per calendar year. This number was applied as the population size. The equation was set with a low (10%) expected prevalence and 95% confidence level.

The CSF samples were obtained only if clinically indicated for each animal via routine cerebellomedullary or lumbar cisternal puncture from anesthetized patients. Unless dog size limited collection volume, an aliquot of 0.2 mL from the collected sample was aseptically placed into a sterile tube for inclusion into the study. The samples were stored at −80°C.

### 2.2 Nucleic acid extraction and PCR

**Figure 1:**
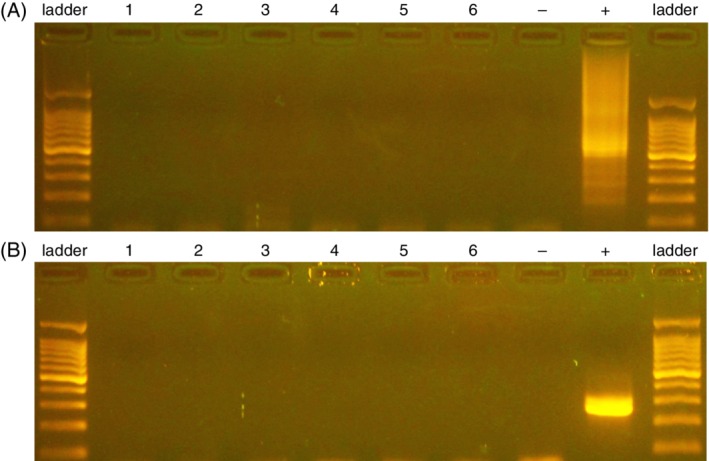
Gel electrophoresis of PCR products. A, Astrovirus PCR products. B, Bornavirus PCR products. Ladder = 100 base pair ladder. Lanes 1‐6 are PCR from canine CSF samples. − = negative control. + = positive control (bottlenose dolphin astrovirus for astrovirus, Parrot bornavirus 4 for bornavirus)

RNA was extracted from 100 μL of supernatant fluid of CSF using a commercial extraction kit (Qiagen RNeasy Kit, Qiagen) according the manufacturer's instructions. The extracted RNA was stored in duplicates as single‐use aliquots at −80°C until PCR amplification. An internal control was performed on each sample to confirm for the presence of 18S (RNA) (Table 1). For amplification of Astroviridae, a OneStep RT‐PCR kit (Qiagen) was used as previously described with primers shown to amplify a both genera, *Avastrovirus* and *mamastrovirus*, of *Astroviridae*, Astr4380F (5′GAYTGGRCNCGNTWYGATGGNA‐CIAT‐3′) and reverse primer Astr4811R (5′‐GGYTTNACCCA‐CATNCCAAA‐3′), targeting conserved regions of astrovirus ORF1b (RNA‐dependent RNA polymerase).^29^, ^30^, ^31^ Bottlenose dolphin astrovirus was used as a positive control (Figure 1A). For amplification of Bornaviridae, primers designed to target a conserved region of the L gene of both genera (5′ GGNATGAGRCARAARYTNTGRAC and 5′ AARTAYTGYTTYTTNCCRTAYTCRTA), *Orthobornavirus* and *Carbovirus*, via PCR were used as previously described.^32^ *Parrot bornavirus 4* was used as a positive control (Figure 1B).

**Table 1:** Cerebrospinal fluid analysis sample results and RNA content

	TNCC/μL	RBC/μL	Protein (mg/dL)	18S
	(x¯, range)	(x¯, range)	(x¯, range)	(x¯, range)
MUE cases (20)	159.6 (8‐1007)	139.24 (0‐4110)	73.6 (27‐293)	22.43 (15.37‐28.14)
Controls (5)	2.8 (1‐5)	2.6 (1‐6)	36 (20‐52)	27.09 (25.82‐28.67)

Abbreviations: MUE, meningoencephalitis of unknown etiology; RBC, red blood cells; TNCC, total nucleated cell counts.

Any identified final amplicons visualized by UV light after gel electrophoresis and bands were to be cut from the gel, extracted using the Qiaquick gel extraction kit (Qiagen, Valencia, California), and submitted to Genewiz (South Plainfield, New Jersey) for sequencing.

## 3 RESULTS

### 3.1 Study population and sample collection

Cerebrospinal fluid was collected from 20 MUE cases and 5 control dogs. The MUE cases included 4 terrier breeds, 4 Malteses, 4 Shih Tzus, 3 Chihuahuas, 2 Poodles, and 1 of each of Pomeranian, Brussels Griffon, and Yorkshire Terrier. They included 10 neutered males, 1 intact male, and 9 spayed females that ranged in age from 2 to 13 years (median 6 years). One of each of Chihuahua, Pomeranian, Yorkshire Terrier, Poodle, and Maltese breeds were included as negative controls. Of the negative controls, 2 were neutered males and 3 were spayed females. Neurolocalization of the MUE cases was intracranial in 12, spinal in 6 and multifocal in 2. Cerebrospinal fluid was collected from the atlanto‐occipital cistern in 19 of the MUE cases and in all control cases. In 1 MUE case, CSF was collected from the lumbar cistern. Prior to CSF collection, 2 of the MUE cases received a single dose of glucocorticoid, and another 2 MUE cases were treated with an antibiotic (Clavamox unknown dose, Zoetis Inc, Kalamazoo, Michigan). Cerebrospinal fluid analysis revealed a pleocytosis in all MUE cases; mean TNCC 159.6 (range, 8‐1007; Table 1). Of note, the 2 cases which received glucocorticoids before CSF analysis had the highest TNCC. Histopathology of CNS tissue performed at the time of necropsy in 3 cases was consistent with GME and 1 case was consistent with NME. The remaining cases were alive at the time of writing or were lost to follow‐up. The control cases were diagnosed with metronidazole intoxication, intervertebral disc disease, Chiari‐like malformation with syringomyelia, and idiopathic cerebellitis. Their age range from 9 months to 13 years (median 9 years).

### 3.2 Nucleic acid extraction and PCR

No amplification of either astroviruses or bornaviruses was identified from the CSF samples of any of the MUE or control cases.

## 4 DISCUSSION

This investigation did not identify any evidence of either astrovirus or bornavirus in the CSF of dogs clinically diagnosed with MUE utilizing reverse‐transcriptase polymerase chain reaction (RT‐PCR). Given these results, we can state with 95% confidence that the prevalence of infection with astrovirus or bornavirus detectable by PCR of CSF is less than 10% in the clinically diagnosed MUE population. Given that MUE is a clinical diagnosis based on magnetic resonance imaging and CSF analysis, obtaining a definitive histopathologic diagnosis of GME, NLE, or NME is often not clinically feasible and biopsy is, in many cases, contraindicated due to the severity of the patient's clinical signs. However, CSF is routinely collected for clinical diagnosis and thus a more available substrate for research in client owned animals.

Previous work has focused on the examination of brain tissue in the quest for putative infectious agents. In a study of formalin‐fixed paraffin‐embedded (FFPE) brain tissue, PCR did not identify the presence of specific DNA viruses in 22 dogs diagnosed with NME, NLE, or GME.^6^ Viral DNA or RNA was not identified utilizing broadly reactive PCR on fresh frozen sections of frontal lobe from 11 and 27 confirmed cases of GME and NME, respectively.^1^ Several possible explanations exist for these results: the use of stored tissue samples which no longer carry the initiating agent of the disease process, the utilized assays lack sensitivity, or, more likely, these viruses were absent in the etiopathogenesis of MUE.

Astroviruses have generally been associated with clinical signs of gastroenteritis and less commonly respiratory disease.^16^, ^17^, ^18^ However, numerous reports across human and veterinary medicine have identified astroviruses as emerging neurotropic pathogens, particularly in immunocompromised transplant recipients humans,^11^, ^12^, ^13^, ^14^, ^16^, ^17^, ^18^, ^33^, ^34^ A novel bovine astrovirus was identified by PCR in the brainstem, cerebellum, and spinal cord of steer diagnosed with an encephalomyelitis and ganglioneuritis of unknown origin.^13^ Frequently diagnosed encephalitis in cattle with unresolved etiology, ESBE, was linked to an astrovirus.^18^ Like GME, NME, and NLE, a definitive diagnosis of ESBE is based on histopathologic results. Investigators utilized RT‐PCR and in situ hybridization on FFPE brain tissue of histologically confirmed ESBE cases to detect astrovirus in 12 of 14 cases (85%).^18^ Most recently, astrovirus was identified by RT‐PCR and in‐situ hybridization in archived CNS samples of newly weaned paraplegic pigs in Hungary.^16^ Given that the initiating etiology for MUE remains to be determined, it is reasonable to consider the possible role of astroviruses in its pathogenesis.

Bornavirus disease is associated with non‐suppurative encephalomyelitis in horses and sheep in endemic areas of central Europe.^35^ There are geographically widespread reports of bornavirus disease in diverse vertebrate hosts including 2 dogs.^19^, ^32^, ^35^ The dogs with bornavirus were both diagnosed with non‐suppurative encephalitis characterized by large perivascular lymphocytic cuffs with varying numbers of macrophages and plasma cells.^19^, ^35^ The GME histologic hallmark is perivascular cuffs composed of mixed lymphoid inflammatory cells, as the canine bornavirus.^25^ Unlike GME but similar to NME and NLE, the lesion distribution of Borna disease in the 2 dogs was predominant in the gray matter but identified throughout the nervous system.^19^, ^25^, ^32^, ^36^ Given these similarities and the current unknown prevalence of bornavirus in the dog, this virus warranted further inquiry.

Several limitations apply to this work. Only a small number of cases were evaluated. The use of 20 clinically diagnosed MUE cases only allows to state with 95% confidence that the prevalence of PCR‐detectable astrovirus in CSF of clinically diagnosed MUE cases is less than 10%.^28^ Secondly, given that CSF samples were obtained antemortem from client‐owned animals, histopathologic confirmation of GME, NME, or NLE was not available for the majority of cases. A confirmed diagnosis of GME or NME was available in 4 affected dogs that were euthanized due to the severity of the disease and whose owners approved a necropsy. Therefore, 16 cases were presumptively diagnosed with MUE. The authors acknowledge that other disease processes, such as lymphoma, could not be definitively excluded. However, obtaining a definitive diagnosis is a common clinical challenge as the risks of obtaining a CNS biopsy often outweigh the benefits in these dogs. More importantly, while CSF is a very clinically applicable sample to collect from client‐owned animals, there is a chance that viral nucleic acids was not available for identification in the small sample collected. In the published human cases, astrovirus capsids were identified in astrocytes, while veterinary reports in swine and cattle primarily identified viral RNA in neurons at the sites of greatest pathology.^13^, ^16^, ^17^, ^33^, ^34^ Similarly, Joest‐Degen bodies, which are typically intranuclear but sometimes intracytoplasmic inclusions, are rarely identified in neurons of the brains of animals diagnosed with bornavirus.^35^ Both astrocytes and neurons are cell types not typically present in CSF. Bornavirus virions are not typically found extracellularly in large numbers, and cell‐to‐cell transmission has been shown.^37^ The assessment of fresh CNS tissue for the presence of astrovirus and bornavirus could prove more fruitful. Although PCR assays designed for diagnostic discovery of the RNA viruses in a variety of host species were utilized, it is possible that the PCR assays did not detect the particular astrovirus or bornavirus in these dogs. Novel astroviruses were identified in the emerging case reports on astrovirus as a neurogenic pathogen. Similarly, novel divergent bornaviruses were sequenced from Australian pythons with neurologic signs.^31^ Intrathecal antibody production was not evaluated as this study focused on the presence of virus in CSF. Serum and CSF antibody titers could be evaluated in affected dogs, though Borna disease virus does to not elicit a strong humoral immune response in naturally infected cats.^38^

In conclusion, no evidence of astrovirus or bornavirus RNA was identified in the CSF of dogs with MUE. This suggests that neither astrovirus nor bornavirus was present in the CSF but could have been present in the CNS parenchyma of dogs clinically diagnosed with MUE, that the organisms were present in lower quantities or were not detectable by the PCR assays, that the infectious agent may have been cleared by the time of testing, or that they are not involved in the etiology of clinically diagnosed MUE. Further investigation of astroviruses and bornaviruses in brain samples collected via biopsy or at the time of necropsy should be considered.

## CONFLICT OF INTEREST DECLARATION

Authors declare no conflict of interest.

## OFF‐LABEL ANTIMICROBIAL DECLARATION

Authors declare no off‐label use of antimicrobials.

## INSTITUTIONAL ANIMAL CARE AND USE OF COMMITTEE (IACUC) OR OTHER APPROVAL DECLARATION

Approved by the University of Florida's IACUC; IACUC Study #201609667. Approval by the College of Veterinary Medicine's Veterinary Hospital Research Review Committee (VHRRC) and a client consent form were not necessary as this is a tissue‐only study.

## HUMAN ETHICS APPROVAL DECLARATION

Authors declare human ethics approval was not needed for this study.
